# Isolation and Characterization of Novel Canine Osteosarcoma Cell Lines from Chemotherapy-Naïve Patients

**DOI:** 10.3390/cells12071026

**Published:** 2023-03-27

**Authors:** Natascha Leitner, Reinhard Ertl, Simone Gabner, Andrea Fuchs-Baumgartinger, Ingrid Walter, Juraj Hlavaty

**Affiliations:** 1 Institute of Morphology, Working Group Histology, University of Veterinary Medicine, Veterinaerplatz 1, A-1210 Vienna, Austria; 2 VetCore Facility for Research, University of Veterinary Medicine, Veterinaerplatz 1, A-1210 Vienna, Austria; 3 Institute of Pathology, University of Veterinary Medicine, Veterinaerplatz 1, A-1210 Vienna, Austria

**Keywords:** canine osteosarcoma, cell line, 3D model, in vitro studies

## Abstract

The present study aimed to establish novel canine osteosarcoma cell lines (COS3600, COS3600B, COS4074) and characterize the recently described COS4288 cells. The established D-17 cell line served as a reference. Analyzed cell lines differed notably in their biological characteristics. Calculated doubling times were between 22 h for COS3600B and 426 h for COS4074 cells. COS3600B and COS4288 cells produced visible colonies after anchorage-independent growth in soft agar. COS4288 cells were identified as cells with the highest migratory capacity. All cells displayed the ability to invade through an artificial basement membrane matrix. Immunohistochemical analyses revealed the mesenchymal origin of all COS cell lines as well as positive staining for the osteosarcoma-relevant proteins alkaline phosphatase and karyopherin α2. Expression of p53 was confirmed in all tested cell lines. Gene expression analyses of selected genes linked to cellular immune checkpoints (CD270, CD274, CD276), kinase activity (MET, ERBB2), and metastatic potential (MMP-2, MMP-9) as well as selected long non-coding RNA (MALAT1) and microRNAs (miR-9, miR-34a, miR-93) are provided. All tested cell lines were able to grow as multicellular spheroids. In all spheroids except COS4288, calcium deposition was detected by von Kossa staining. We believe that these new cell lines serve as useful biological models for future studies.

## 1. Introduction

Osteosarcoma, a malignant tumor of mesenchymal origin, is the most frequent primary bone malignancy in dogs [1,2]. Despite improvements in survival rates, current treatment modalities are still inefficient due to the relative rareness of this disease, insufficient understanding of the osteosarcoma biology and, above all, its metastatic behavior [2,3,4,5,6]. To overcome this obstacle, the use of well-characterized models in osteosarcoma research is essential.

Next to patient-derived xenografts and organoids, tumor-derived cell lines represent an available, useful model for basic in vitro and in vivo research and preclinical studies on osteosarcoma growth and biology [7,8,9,10]. Several osteosarcoma cell lines have been successfully isolated from human and canine tumors [11,12,13,14,15,16]. These cells were used to detect essential details of the metastatic properties of osteosarcomas, paving the way for a better understanding of their biological properties and putative treatment modalities [5,14,15,17,18,19].

As osteosarcomas represent a very heterogeneous malignancy and the number of available canine osteosarcoma cell lines is limited, their further de novo isolation and characterization are necessary to span the variability of the naturally occurring tumor. In addition, the biological characteristics of canine and human osteosarcomas are similar with; canine osteosarcoma models ideally suited for translational research. Therefore, a deeper understanding about this tumor in humans can be obtained through canine osteosarcoma research [20,21,22].

In the present study, we isolated novel canine osteosarcoma cell lines from chemotherapy-naïve patients and compared them with the commercially available and well-characterized D-17 canine osteosarcoma cell line. Besides their basic phenotypic and biological characterization, cell lines were analyzed immunohistochemically (for vimentin, cytokeratin, ALPL, p53, KPNA2) and were checked for their ability to grow as multicellular tumor spheroids. We further provide gene expression analysis of selected genes encoding for cellular immune checkpoints (CD270, CD274, CD276), proteins with kinase activity (MET, ERBB2), and genes linked to metastatic potential (MMP-2, MMP-9), as well as selected long non-coding RNA (MALAT1) and microRNAs affecting cell migration and invasion (miR-9, miR-34a, miR93).

## 2. Materials and Method

### 2.1. Clinical Material, Cell Isolation and Cultivation

Tissue samples from primary canine osteosarcoma tumors (animals without previous tumor treatment history) collected after standard therapeutic intervention were obtained from the VetBioBank/VetCore Facility of the University of Veterinary Medicine, Vienna, in accordance with all ethical rules and legal standards of the University of Veterinary Medicine, Vienna [23]. All samples were classified according to the histological classification of the WHO and graded according to the grading system of Loukopoulos and Robinson [24,25].

To isolate the cells, tumor samples were washed in sterile DPBS supplemented with 2% antibiotic–antimycotic solution (all from Sigma-Aldrich, St. Louis, MO, USA), cut into small pieces and transferred into wells of a 6-well plate (TC plate 6 well standard, F; Sarstedt, Nümbrecht, Germany). After a short adhesion phase (5–10 min) to avoid floating of the tumor pieces in the cultivation medium, DMEM high-glucose medium supplemented with 10% fetal calf serum (FCS), 1% antibiotic–antimycotic solution (all from Sigma-Aldrich), and 1% L-glutamine (Biowest, Riverside, MO, USA) was added. Cells growing out from the tumor pieces were harvested using 0.05% trypsin (Trypsin-EDTA, BioWest). The COS3600, COS3600B, and COS4074 cell lines were established by performing serial passaging (split ratio 1:3 or 1:4) whenever they reached 80–90% confluency. Cells were cultured for at least 20 passages before entering the characterization process, except for COS4074 cells, where the characterization started at passage 17, representing 5–6 month of continuous COS4074 cell cultivation. The isolation of COS4288 cells was carried out in a similar manner, as recently described in [26].

The D-17 cell line was obtained from ATCC (CCL-183^TM^; Manassas, VA, USA) and cultured in MEM (PAN Biotech, Aidenbach, Germany) supplemented with 10% fetal calf serum, 1% antibiotic–antimycotic solution (both from Sigma-Aldrich), and 1% L-glutamine (Biowest, Riverside, MO, USA).

All culturing was carried out in an incubator under standard conditions at 37 °C in a humidified atmosphere containing 5% CO_2_. At various passage numbers, cells were tested for mycoplasma contamination using PCR as described by Vojdani [27].

### 2.2. Cell Line Authentication (STR Analysis)

The original tumors as well as their respective established cell lines were authenticated by examining short tandem repeats (using STR analysis) in 22 loci. Microsatellite analysis was performed by a commercial laboratory accredited according to DIN EN ISO 17025:2018 (Laboklin, Bratislava, Slovak Republic). Nomenclature is based on ISAG comparison test 2006 standards. Cell line integrity was calculated as described in Lin [28].

### 2.3. Growth Kinetic Analysis

Cells seeded in 6-well plates (Sarstedt) were trypsinized at regular time intervals and the cell amount was determined by an automated cell counter (COUTNESS, Invitrogen, Carlsbad, CA, USA). Population doubling time (DT) was calculated using the following formula:

doubling time = cultivation duration × log(2)/log(final cell count) − log(initial cell count)

(https://www.doubling-time.com/compute.php, accessed on 5 July 2021).

### 2.4. Soft Agar Colony Formation Assay

The soft agar colony formation assay was performed as described [26]. Colony formation was evaluated after 21 days for COS3600B, COS4288, and D-17 and after 56 days for COS3600 and COS4074 cells. Pictures were taken using a light microscope (Zeiss Observer Z1, Oberkochen, Germany).

### 2.5. Wound Closure Assay (Scratch Assay)

For measurement of cell migration into a wound region, the IncuCyte^®^ 96-well scratch wound cell migration and invasion system (Sartorius Austria GmbH, Vienna, Austria) was used. Briefly, the following cell amounts were seeded per well of the IncuCyte^®^ Imagelock 96-well Plate: COS3600—3 × 10^4^; COS3600B—1.5 × 10^4^; COS4074—4.5 × 10^4^; COS4288—2 × 10^4^; D-17—1 × 10^4^. A wound (scratch) was created using the IncuCyte^®^ WoundMaker device according to the manufacturer’s recommendations. Wound closure was monitored using a Nikon ECLIPSE Ti2-e microscope equipped with a Nikon DS-Qi2 Camera (Nikon GmbH, Vienna, Austria). Brightfield pictures were taken at 10× magnification (pixel size (x/y): 0.72 µm) using a 3 h time series interval. The acquisition pipeline was scripted with the JOBS module of the NIS Elements acquisition software (Nikon GmbH). Based on automated well recognition, 2 × 2 stitched images (10% blending) were acquired at the well center for each time point. Images were analyzed using Fiji software [29]. Images were processed with a Gaussian filter (s = 2), followed by a Top Hat filter (r = 25). Subsequently, the wound area was measured based on threshold segmentation. Data represent the mean from six replicates.

### 2.6. Cell Culture Insert Migration and Invasion Assay

The migration and invasion potential of osteosarcoma cells was measured using a 24-well plate and polycarbonate cell culture inserts with a pore size of 8 µm (ThermoFisher Scientific, Waltham, MA, USA) as described in Justus, with minor modifications [30].

To assess the migration capacity, 1 × 10^5^ cells/0.5 mL/cell culture insert was seeded in a growth medium with the FCS concentration reduced to 1%. The respective well of the plate was filled with 0.75 mL of the normal growth medium (10% FCS content) and cultivated for 24 h.

To assess the invasion potential, cell culture inserts were coated with 100 µL of Corning^®^ Matrigel^®^ basement membrane matrix (Sigma-Aldrich) diluted to the final concentration of 250 µg/mL in coating buffer (0.01 M Tris pH8, 0.7% NaCl). After two hours of incubation at 37 °C, excessive liquid was carefully removed from the insert and 1 × 10^5^ cells/0.5 mL/insert was seeded in a growth medium with the FCS concentration reduced to 1%. Again, the respective wells of the plate were filled with 0.75 mL of the normal growth medium (10% FCS content) and cultivated for 24 h.

For both migration and invasion assays, non-migrating/non-invading cells were removed from the upper side of the insert using a moistened cotton swab. The inserts containing migrated/invaded cells were fixed in 70% ethanol for 10 min at room temperature. Excessive ethanol was removed from the upper surface of the insert using a dry cotton swab and inserts were air-dried. Staining was performed using 0.2% crystal violet solution (in 2% ethanol) for 10 min at room temperature. After washing in distilled water, inserts were air-dried, and pictures were taken using a light microscope (Zeiss Observer Z1). The number of migrating/invading cells were counted in five independent microscopic fields. The invasion index percentage was calculated using the following formula [31]:

invasion index % = (number of invading cells/number of migrating cells) × 100%

### 2.7. Spheroid Formation Assay

Spheroids were grown as described [26]. In short, 2 × 10^4^ of D-17 cells or 4 × 10^4^ of COS4288 cells were seeded per well of a 96-well low attachment plate (96 w Brand plates U inert Grade clear, Brand GmbH, Wertheim, Germany) and cultivated for up to 21 days. For COS3600, COS3600B, and COS4074 cells, different cell amounts ranging from 1 × 10^4^ to 8 × 10^4^ cells per well were tested to find the optimal seeding density. Formation of spheroids was monitored microscopically (Zeiss Observer Z1). Half of the respective medium volume was changed every second day. On day 21, spheroids were harvested by sedimentation, washed with DPBS and subjected to histochemical analysis as described below.

### 2.8. Histochemical and Immunohistochemical Analyses

Spheroids were fixed in 4% neutral buffered formaldehyde (24 h at room temperature). Formaldehyde was carefully removed from the sedimented pellet, overlaid with Histogel^®^ (Richard-Allan Scientific, Kalamazoo, MI, USA), and subsequently paraffin-embedded by means of an automatic Tissue-Tek VIP embedding device (Sakura Finetek Europe B.V., Umkirch, Germany). Sections of 3 µm thickness were cut and either stained with hematoxylin and eosin (H&E) according to Romeis for morphological analyses or further processed for histochemical analyses [32].

To detect extracellular calcium deposition, von Kossa staining was performed as described in Mulisch [33]. In short, spheroid sections were incubated in 5% silver nitrate solution (Carl Roth, Karlsruhe, Deutschland) for 20 min in direct sunlight, washed in distilled water, and fixed for in 5% sodium thiosulphate solution (Carl Roth) for 2 min. Finally, slides were washed with distilled water, counterstained with nuclear fast red (aluminium solution (0.1%, Waldeck, Münster, Germany)), dehydrated, and mounted using DPX medium (Fluka, Buchs, Switzerland). Evaluation of sections was performed using light microscopy (BX53, Olympus, Shinjuku, Japan).

To detect acidic polysaccharides such as glycosaminoglycans of cartilage-like matrix, Alcian blue staining and Safranin O staining were performed as described in Mulisch [33]. For Alcian blue staining, spheroid sections were incubated in 3% acetic acid for 3 min followed by 30 min incubation in 1% Alcian blue 8GX solution (*w*/*v* in 3% acetic acid, Sigma). Following a quick washing step in 3% acetic acid and distilled water, nuclear counterstaining was performed with nuclear fast red solution (Waldeck GmbH, Münster, Deutschland).

For Safranin O staining, spheroid sections were incubated in hematoxylin solution according to Weigert (Fluka) for 1 min, washed in 80% ethanol and distilled water, and incubated in 0.1% Light Green solution (Fluka, *w/v* in distilled water) for 2 min. Subsequently, sections were washed in 1% acetic acid (Sigma) for 2 min and stained with a 0.1% Safranin O solution (*w*/*v* in distilled water, Sigma). To finalize both stainings, slides were washed with distilled water, dehydrated, and mounted with DPX medium (Fluka). Evaluation of the sections was performed using light microscopy (BX53, Olympus).

Cells for immunohistochemical analyses were grown on 4-well glass chamber slides (Lab-Tek II Chamber Slide System, ThermoFisher Scientific, Waltham, MA, USA). Immunofluorescent detection of cytokeratin and vimentin as well as immunodetection of alkaline phosphatase (ALPL) and karyopherin α2 (KPNA2) was carried out as described [26].

For immunohistochemical detection of p53, cells were first fixed in 4% neutral buffered formaldehyde (10 min at room temperature). This was followed by cell permeabilization by incubation in 0.2% TritonX-100 (Merck) in PBS (15 min at 4 °C), blocking of endogenous peroxidases (0.6% H_2_O_2_ in methanol; 15 min at room temperature), antigen retrieval (30 min in Tris-EDTA pH 9, in a steamer), and protein blocking in 1.5% normal goat serum in PBS (30 min, room temperature). Afterwards, samples were incubated with the monoclonal mouse anti-p53 [clone PAb122] antibody (cat.# ADI-KAM-CC002-D, Enzo Life Sciences, Lausen, Switzerland; dil. 1:1000) overnight at 4 °C. Signals were detected with the BrightVision Poly-HRP-anti-mouse secondary system (Immunologic, Duiven, Netherlands) using DAB-solution (Quanto, ThermoFisher) as a substrate. Lastly, samples were counterstained with hematoxylin (Epredia, Richard Allan Scientific, Microm International, Walldorf, Germany), mounted using DPX medium (Fluka), and evaluated using light microscopy (Olympus BX53).

### 2.9. Reverse Transcription Quantitative PCR Analysis

Osteosarcoma cells were lysed in TRI reagent (Zymo Research, Irvine, CA, USA) and total RNA was extracted with the Direct-zol RNA Miniprep Kit (Zymo Research). For mRNA quantification, 1 µg total RNA was used for cDNA synthesis with the High Capacity cDNA Reverse Transcription Kit (Thermo Fisher, Waltham, MA, USA) according to the manufacturer’s protocol. No-RT controls (without enzyme) were included with each sample to monitor the detection of contaminating DNA. RT-qPCR primer details are listed in Supplementary Table S1 [34,35,36]. Each RT-qPCR was performed in duplicate using a 20 µL reaction volume containing 1x HOT FIREPol EvaGreen qPCR Mix Plus ROX (Solis BioDyne, Tartu, Estonia), 200 nM of each primer, and 20 ng cDNA in an AriaMx Real-time PCR System (Agilent, Santa Clara, CA, USA) with the following temperature profile: 95 °C for 12 min, 40 cycles of 95 °C for 15 sec and 60 °C for 1 min, followed by a melting curve analysis (60–95 °C). OAZ1, RPL8, RPL27, and RPL32 were included as potential reference genes (RGs) and measured in all experimental samples. Based on the results of the RefFinder analysis tool, the two most stably expressed genes, OAZ1 and RPL27, were selected for normalization [37]. Target gene expression values were normalized to the geometric mean of both RGs and expression levels were calculated using the ΔCt method [38]. For quantification of miRNAs, 200 ng total RNA was used for miRNA polyadenylation and reverse transcription with the MystiCq microRNA cDNA Synthesis Mix (Sigma-Aldrich). Target miRNA sequences were obtained from miRBase database release 22.1 and primers were designed with miRprimer software v2 (Supplementary Table S1) [39,40]. RT-qPCR for miRNAs was carried out in 20 µL reactions which included 1x MystiCq microRNA SYBR Green qPCR Ready Mix Low ROX (Sigma-Aldrich), 200 nM MystiCq Universal PCR Primer (Sigma-Aldrich), 200 nM assay-specific forward primer, and 1 ng cDNA using the following temperature profile: 95 °C for 2 min, 40 cycles of 95 °C for 5 s and 60 °C for 30 s, and a melting curve step (60–95 °C). Two endogenous control RNAs were included for normalization; U6 snRNA, a commonly used normalizer, and cfa-miR-103, the canine ortholog of miR-103a-3p which was previously found to be a suitable reference for miRNA expression studies in human osteosarcoma tumors [41]. The RefFinder tool identified cfa-miR-103 as the more suitable reference, which was then used for normalization. The calculation of RNA expression levels was performed as described above.

### 2.10. Statistical Analysis

Statistical analyses (unpaired t-test with Welch’s correction) were performed with GraphPad Prism 8.4.3 (GraphPad Software, San Diego, CA, USA). A *p*-value < 0.05 was considered significant.

## 3. Results

### 3.1. Cell Isolation and Morphology

The material used for cell line establishment originated from primary bone tumors in adult animals. The COS3600 and COS3600B cells were isolated from a 14 y old male dog patient of mixed breed with a tumor located at the humerus that had been diagnosed as osteoblastic osteosarcoma grade III. The COS4074 cells were isolated from a tumor diagnosed as chondroblastic osteosarcoma grade II located at the humerus of a 13.5 y old male bullmastiff. COS4288 cells originated from an osteoblastic osteosarcoma grade III tumor located on the femur of a female Labrador retriever patient [26]. All animals were chemotherapy naïve. Although basic characterization of the COS4288 cells was recently published, we provide the COS4288 characteristics along with the characteristics of the newly established COS3600, COS3600B, and COS4074 cell lines to maintain the study’s coalescence [26].

**Figure 1:**
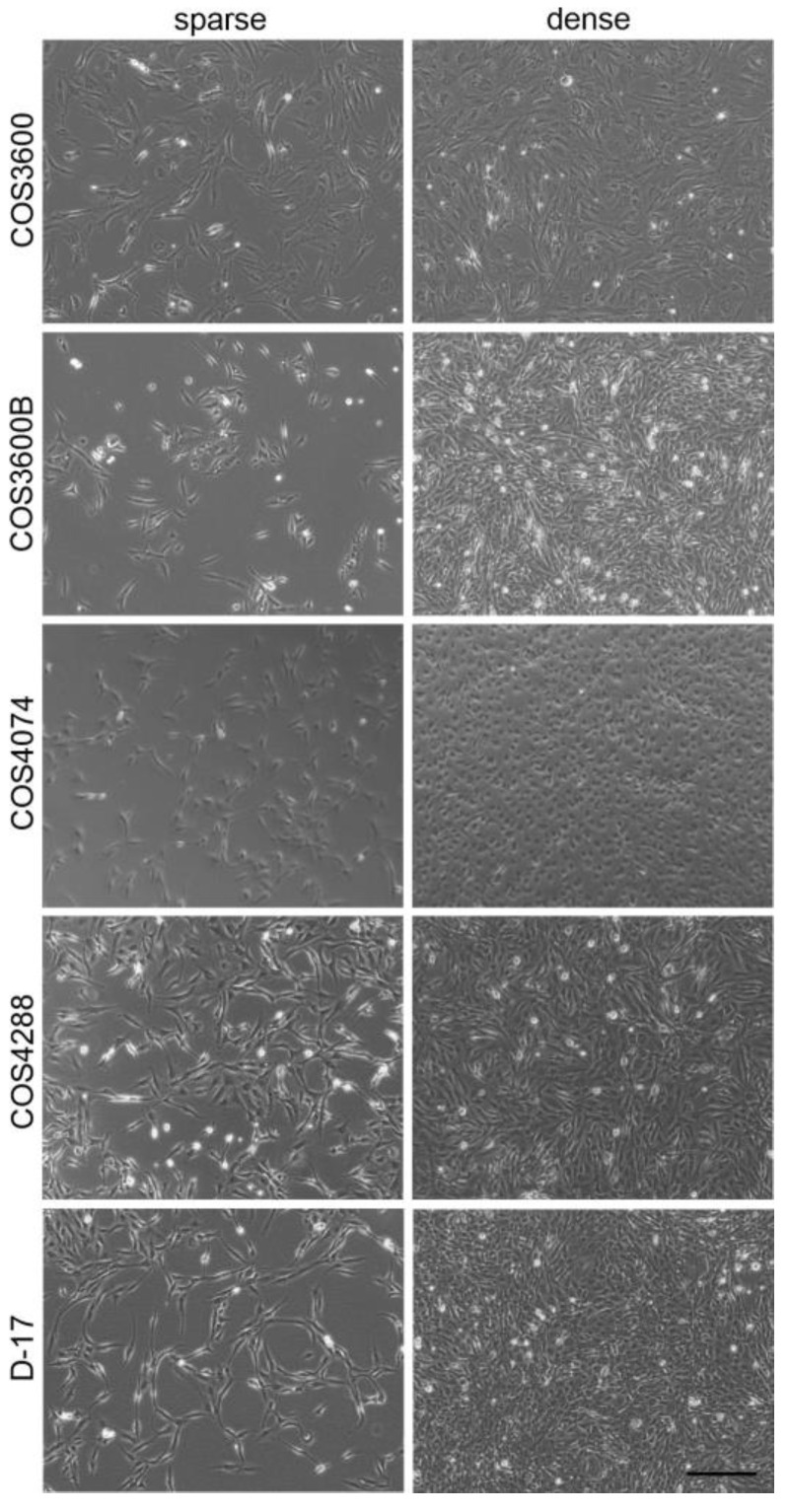
Cell morphology of isolated canine osteosarcoma cells and reference D-17 cells grown under 2D culture conditions. Pictures of sparse and dense cell populations are shown. Scale bar—200 µm.

In the early step of COS3600 cultivation (approx. 2 weeks after initiation), two different cell sub-populations were identified: the original COS3600 cells (Figure 1), with spindle-shaped morphology and only sporadic deltoid-shaped cells; and COS3600B cells (Figure 1), with numerous cobblestone-like cells and spindle-shaped cells smaller than those observed in COS3600. The COS3600 cells grew substantially slower compared with COS3600B cells. With increased cultivation time, COS3600 cells became more resistant to trypsinization, demanding longer incubation times for cell detachment compared with COS3600B. The COS4074 cells (Figure 1) formed small deltoid and polygonal cells with rare spindle-shaped cells and required prolonged trypsinization time as well. COS4288 cells (Figure 1) consisted of a uniform, deltoid-shaped cell population [26].

Before considering newly isolated cells a cell line, and before entering the characterization process, cells had been cultured for at least 20 passages, except for COS4074 cells, whose characterization started at passage 17, representing 5–6 months of continuous culture and having undergone freezing-thawing cycles in liquid nitrogen without affecting their growth potential. In the time span of 5–6 months, COS3600B and COS4288 reached passage numbers of over 50, whereas COS3600 and COS4074 reached passage number 35 and 25, respectively. All cells tested negative for mycoplasma.

### 3.2. Authentication of the Cell Lines

To authenticate the established cell lines, we examined the STR status of 22 canine microsatellites encompassed in the comparison test for animal DNA testing of the International Society for Animal Genetics (Table 1) and compared them with the STR profile of the original tumors (Supplementary Table S2). Analysis revealed that the genetic background of COS3600/COS3600B, COS4074, and COS4288 cell lines came from three different animals. The sex determination using amelogenin analysis was in accordance with the patient history. Isolated cell lines showed a matching STR profile with the corresponding original tumors, with evaluation values of 0.95 (COS3600), 0.84 (COS3600B), 0.95 (COS4074), and 0.87 (COS4288). Detailed STR analysis of COS3600 and COS3600B cells confirmed the shared origin of both cell lines (35 identical alleles, 7 different alleles; evaluation value 0.83) and excluded cross-contamination with other cell lines (evaluation value lower than 0.8).

### 3.3. Growth Kinetic Analysis

Growth rates were assessed based on cell counts measured over time. Calculated population doubling time (DT) differed substantially between the cell lines. D-17, COS3600B, and COS4288 cells showed a DT of 19, 22, and 32 h respectively, whereas COS3600 and COS4074 revealed extremely long DTs of 139 and 426 h, respectively. The difference in DT was most notable between COS3600 and COS3600B cells, despite their shared tumor origin.

### 3.4. Anchorage-Independent Growth in Soft Agar

**Figure 2:**
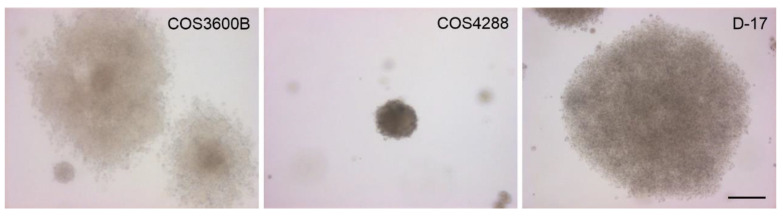
Anchorage-independent cell growth and colony formation in a semisolid medium. COS3600B, COS4288, and D-17 cells formed colonies within 21 days of culture. Scale bar—200 µm.

Anchorage-independent growth as a hallmark of carcinogenesis was further investigated in the respective cell lines using the soft agar colony formation assay. After 21 days of cultivation, distinct colonies of different sizes and cell densities were formed by COS3600B, COS4288, and D-17 cells (Figure 2) [26]. Colonies originating from COS3600B and D-17 cells were bigger compared with those from COS4288 (~1 mm vs. ~250 µm) and displayed a diffuse border that was more pronounced in COS3600B cells. Neither colonies nor cell clusters were found in COS3600 and COS4074 cells, despite a prolonged cultivation time of 56 days.

### 3.5. Cell Migration and Invasion Analyses

The ability to migrate and/or invade through an artificial basement membrane was analyzed as an indicator of the metastatic potential of the tumor cells.

**Figure 3:**
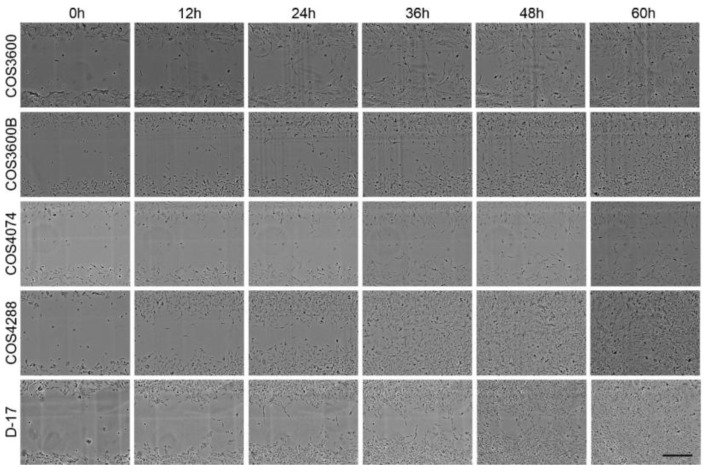
Exemplary pictures of the wound closure assay of isolated canine osteosarcoma cells and reference D-17 cells at different time points. Only COS4288 and D-17 cells fully closed the scratch after 60 h. Scale bar—400 µm.

**Figure 4:**
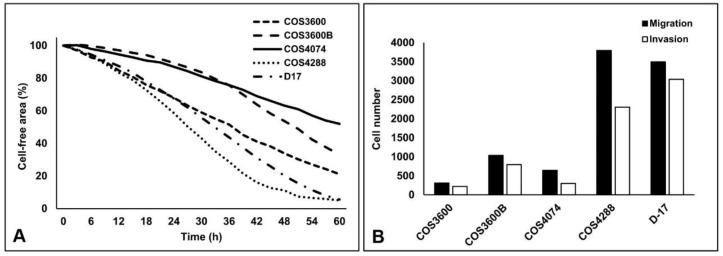
Graphical presentation of the wound closure assay (**A**) as well as of the cell culture insert-based migration and invasion assays (**B**) of isolated canine osteosarcoma cells and reference D-17 cells. The data shown in (**A**) were obtained from three independent biological replicates, whereas each biological replicate was done in a duplicate. The degree of variability between replicates for each cell line was as follows: COS3600 1.6–6.4%, COS3600B 0.2–5.5%, COS4074 1.0–7.8%, COS4288 1.3–5.9%, D-17 0.8–5.0%. The indicated lines represent mean values. The data shown in (**B**) were obtained in a single experiment.

First, migration capacity was measured over 60 h via a wound closure assay (Figure 3). COS4288 cells revealed the highest migration ability, as the scratch was closed as early as 48 h post wounding. The migration capability of COS3600 and COS3600B cells was similar with a small, but still visible, scratch after 60 h. The cell-free area after 60 h was biggest in COS4074 cells, clearly indicating the lowest migration capacity in these cells (scratch visible even after 72 h—data nor shown). D-17 cells fully closed the scratch within 60 h. Detailed quantification of the cell-free area in all cell lines over the tested 60 h period is summarized in Figure 4A.

**Figure 5:**
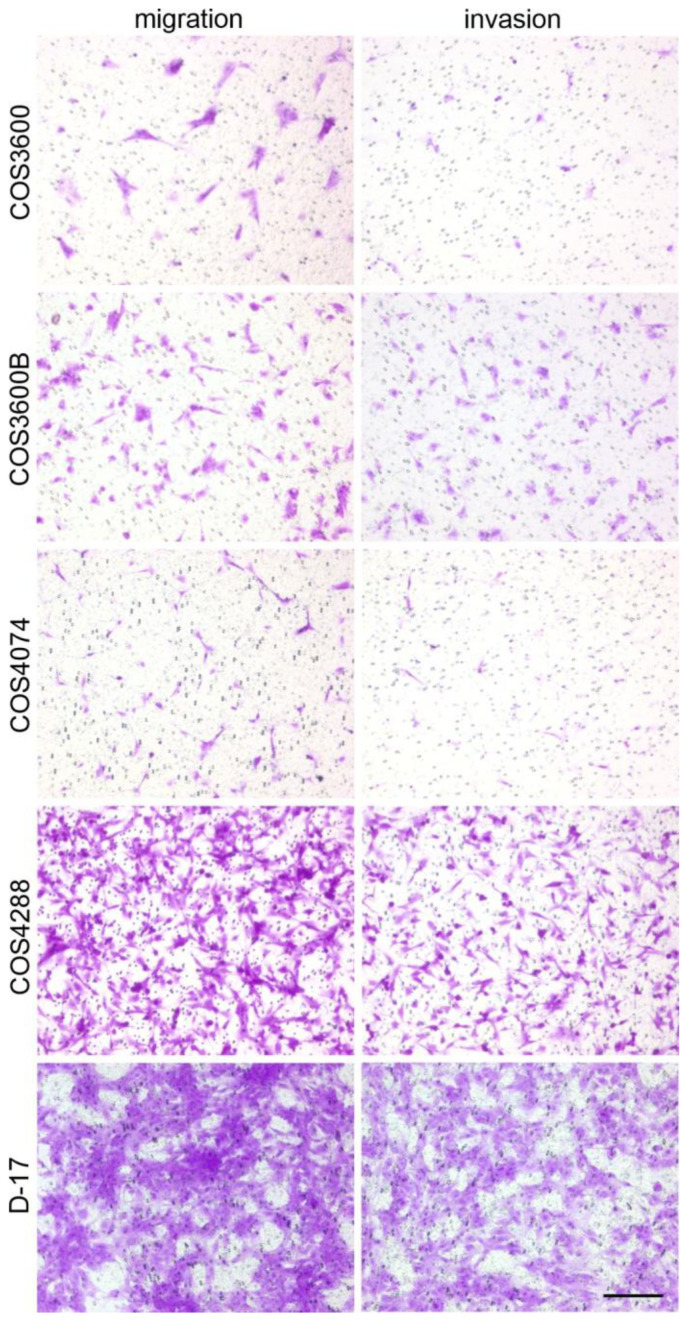
Exemplary pictures of the cell culture insert-based migration and invasion assays of crystal violet-stained isolated osteosarcoma cells and reference D-17 cells. Scale bar—200 µm.

To further support these observations, an alternative cell culture insert-based migration assay was employed, where the number of cells migrating through a membrane with 8 µm pores was estimated 24 h after cell inoculation. Despite identical seeding density, there were differences in the number of cells crossing the membrane. Similar to that described above, the migration potential of COS4288 was superior to that of COS3600B, COS3600 and COS4074 cells, as documented by quantitative analysis (Figure 4B) and a representative picture of stained inserts (Figure 5). Based on observations from both the wound closure and the insert-based assay, the migration capacity of tested osteosarcoma cells decreases as follows: COS4288 > D-17 > COS3600/COS3600B > COS4074.

To mimic invasion processes observed during the metastatic spread of tumors, cells were analyzed for their capacity to cross a Matrigel^®^ layer resembling a basement membrane matrix on the cell culture insert (Figure 4B and Figure 5). As invasive capability is a complex process, high migration rate is not sufficient to ensure also high invasive potential of the cells. Indeed, by comparing migration and invasion abilities, the invasion index was calculated as follows: 71% (COS3600), 77% (COS3600B), 46% (COS4074), 61% (COS4288), and finally 87% (D-17).

### 3.6. Immunohistochemical Analyses on Cell Monolayers

**Figure 6:**
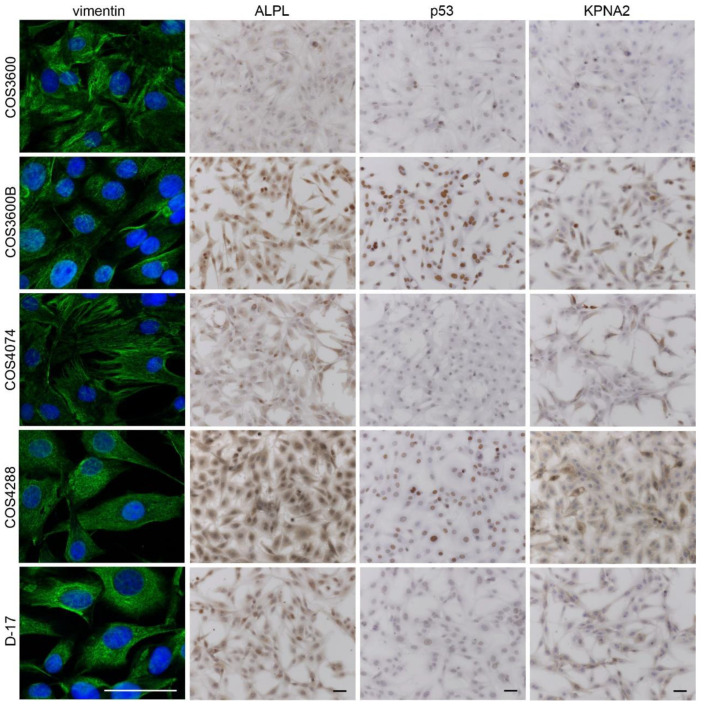
Exemplary pictures of immunofluorescent staining (vimentin—green) and immunohistochemical staining (ALPL, p53, and KPNA2—brown) of isolated canine osteosarcoma cells and reference D-17 cells. Nuclei were counterstained with DAPI (vimentin) or hematoxylin (ALPL, p53, and KPNA2). Scale bar—50 µm.

To further confirm the origin of the isolated cell lines, immunofluorescent staining for the mesenchymal marker vimentin and epithelial marker cytokeratin was performed. All cells stained positively for vimentin (Figure 6) and stained negatively for cytokeratin.

Alkaline phosphatase staining was found in all isolated COS cells as well as in D-17 cells (Figure 6), with variable staining intensity. In COS3600B and COS4288 cells, strong ALPL expression was detected in all cells. In COS3600, COS4074, and D-17 cells, both low and high ALPL expressing cells were detected.

The p53 protein was detected in the nuclei of all analyzed cell lines (Figure 6). Strong nuclear positivity was seen in the majority of COS3600B cells, while COS4288 revealed a mixed population consisting of strongly and moderately positive cells. COS3600, COS4074, and D-17 cells expressed p53 only at low levels.

The KPNA2 protein was detected in the nuclei and in cytoplasm of all tested cell lines (Figure 6). Strong expression was observed in COS3600B, COS4288, and D-17 cells. In COS3600 and COS4074 cells, the expression levels were low and intermediate, respectively.

### 3.7. Reverse Transcription Quantitative PCR Analysis

**Figure 7:**
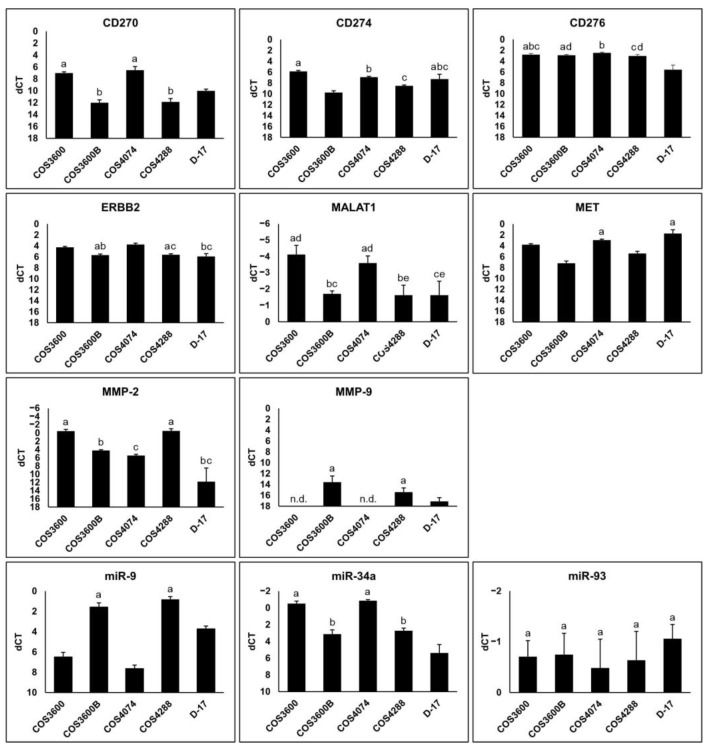
Graphical presentation of RT-qPCR results of selected genes and miRNAs of isolated canine osteosarcoma cells and reference D-17 cells. Bars depict the differences between the respective gene and the reference genes (dCT value); whiskers represent standard deviation. An inverted *y*-axis was chosen to illustrate higher RNA and miRNA levels with higher bars. Differences between cell lines are significant (*p* < 0.05) if they do not feature the same letter. n.d.—not detected.

Basal gene expression for matrix metalloproteinase 2 and 9 (MMP-2 and MMP-9), MET receptor tyrosine kinase (MET), erb-b2 receptor tyrosine kinase (ERBB2), tumor necrosis factor receptor superfamily member 14 (CD270, HVEM), programmed death-ligand 1 (CD274, PD-L1), and cluster of differentiation 276 (CD276, B7-H3) proteins, along with the expression levels of metastasis-associated lung adenocarcinoma transcript 1 (MALAT1) long non-coding RNA and microRNAs miR-9, miR-34a, miR-93 were measured in the new cell lines and D-17 cells (Figure 7).

In general, genes were differentially expressed in the analyzed osteosarcoma cell lines (Figure 7). CD270 expression was highest in COS3600 and COS4074 cells, whereas COS3600B cells revealed the lowest CD274 levels. CD276 expression was significantly lower in D-17 cells compared to all COS cells. ERBB2 and MALAT1 mRNA levels were highest in COS3600 and COS4074 cells. MET expression was significantly higher in COS4074 and D-17 cells. While MMP-2 expression was especially high in COS3600 and COS4288 cells, MMP-9 mRNA was not detectable in COS3600 and COS4074 cells. MicroRNA analysis revealed significantly lower miR-9 but higher miR-34a levels in COS3600 and COS4074 cells. Levels of miR-93 were similar in all tested cell lines.

### 3.8. Spheroid Formation

The ability to form spheroids was confirmed for COS3600, COS3600B, and COS4074 cells. COS4288 spheroid formation has previously been partly described [26]. In COS3600 and COS3600B cells, distinct signs of 3D growth were observed after 24 h of cultivation: a dark central part corresponding to the bulky spherical cell structure in the middle was surrounded by a light-colored corona. On the contrary, COS4074 cells formed irregular structures first, which became round and organized into a spheroid around day seven.

The optimal cell seeding density for production of spheroids cultivated for 21 days differed between these cell lines. Seeding of 8 × 10^4^ cells per well was found to be optimal for forming COS3600 and COS4074 spheroids, as lower cell amounts produced substantially smaller spheroids. For COS3600B cells, 2 × 10^4^ cells per well was optimal as higher cell numbers resulted in only slightly larger spheroids. In addition, these larger spheroids often showed signs of disintegration with a release of cells or cell clumps from the spheroid.

**Figure 8:**
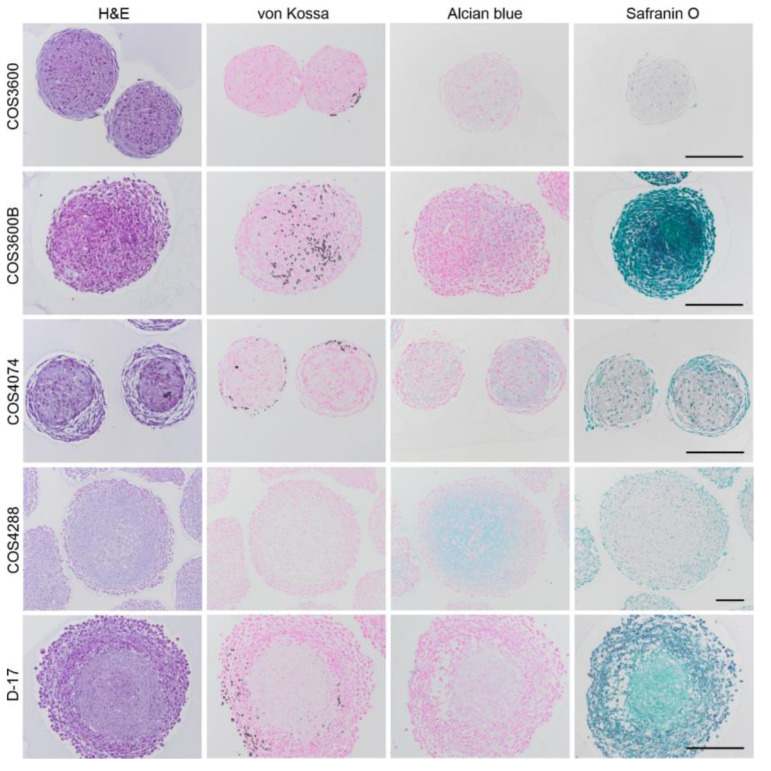
Histological and histochemical staining of 3D spheroids of isolated canine osteosarcoma cells and reference D-17 cells. Hematoxylin and eosin staining revealed a concentric organization consisting of two (COS cells) or three (D-17) zones with different cell densities and organization. In all cell lines except COS4288 cells, calcium deposition (black) was detected by von Kossa staining. Alcian blue-stained glycosaminoglycans (blue) were absent in COS3600 and D-17 cells, scant in COS3600B and COS4074 cells, and prominent in the center of COS4288 cells. No Safranin O staining was detected in all cell lines. Scale bar—200 µm.

Hematoxylin and eosin staining of spheroid sections revealed a concentric organization comprised of two (all COS cells) or three (D-17) zones with different cell densities and organization (Figure 8). In COS3600 and COS4074 spheroids, a central zone with round cells was surrounded by an outer zone consisting of loosely arranged fibroblast-like cells. Consisting of slowly growing cells, these spheroids were smaller (approx. 200 µm) compared with other analyzed spheroids; furthermore, the central zone did not show signs of necrosis and contained less extracellular matrix. In COS3600B spheroids, the cell shape was homogenous throughout the whole spheroid and the zonal distribution was based on cell density only, with a higher cell density in the central zone. Despite their size (approx. 400 µm) and cell density, the central zone did not show signs of necrosis. In COS4288 spheroids, a central zone with round cells of chondroid appearance in high amounts of basophil matrix was surrounded by an outer layer of spindle-shaped and polygonal cells. In D-17 spheroids, central, intermediate and outer zones (necrotic, quiescent, and proliferative) were clearly defined as previously described [26,42].

### 3.9. Histochemical Analyses of 3D Spheroids

Induced by the 3D growth, cells formed mineralized nodules detectable with von Kossa staining in all but COS4288 spheroids (Figure 8). However, their quantity and the location within the spheroid were different. Calcium depositions were located in the outer zone of COS3600 and COS4074 spheroids, and in the outer and intermediate zone of D-17 spheroids, whereas they were distributed within the whole COS3600B spheroids.

In the next step, we assessed the presence of extracellular matrix in the spheroids. In COS4288 spheroids, distinct Alcian blue positive extracellular matrix was localized in the central zone (Figure 8). Only weak Alcian blue staining was seen in COS3600B and COS4074 spheroids and no Alcian blue positive areas were observed in COS3600 and D-17 spheroids. Safranin O staining was negative in all tested samples.

## 4. Discussion

Canine osteosarcoma is a highly heterogeneous, aggressive bone malignancy, affecting mostly large breeds in middle-aged or older dogs [43,44,45]. Indeed, each of the four newly generated and characterized cell lines demonstrated different biologic behavior indicating the necessity of various (patient specific) tumor cell lines for in vitro testing.

COS3600B and COS4288 cells possessed a DT similar to those typical for other canine osteosarcoma cell lines, e.g., D-17 and Abrams [46]. However, the estimated DT may differ due to cultivation condition and methodology, as can be seen by comparing data for Abrams cells obtained by Maeda [46] and Wilson-Robles [16]. The DT of COS3600 and COS4074 was extremely extended and, to our best knowledge, does not have analogy among published osteosarcoma cell lines. Surprisingly, COS3600 and COS3600B cells, both originating from the same tumor, revealed a marked DT difference that might be related to the intra-tumoral heterogeneity. Despite known limitations in cancer cell line identification by short tandem repeat profiling, we performed this analysis on all four cell lines and confirmed shared origin of COS3600 and COS3600B cells while concurrently demonstrating separate genetic background of COS4288 and COS4074 cells [47,48].

Proliferation of cells other than those of hematological origin without attachment to a firm base (anchorage-independent cell growth) is considered as one of the signs of malignant transformation [49]. This feature was previously described in several canine and human osteosarcoma cell lines, including D-17 and COS4288 cells [14,26,50]. Our experiments confirmed the ability for anchorage-independent cell growth of COS4288 and D-17 cells. Regarding the other COS cells, only COS3600B cells produced visible, multicellular colonies in soft agar within 21 days. The colony formation was absent in COS3600 and COS4074 cells even after prolonged cultivation time of 56 days. This observation agrees with previously published data showing that not all tumor cells are able to form colonies in the soft agar and the cells with increased stemness potential have a higher capability to produce colonies [51,52,53]. We know yet nothing about the stemness potential of isolated COS cells, so we cannot attribute the lack of colonies to the absence of cancer stem cells in COS3600 and COS4074 cells. However, soft agar colonies producing cells (COS3600B, COS4288, and D-17) revealed high migration and/or invasion potential in our experiments. We also believe that the cell doubling time and requirement of a certain “minimal seeding density” also strongly affects the capability to form these colonies. Seeding the COS3600 and especially COS4074 cells at a low density (e.g., split ratio more than 1:4) can dramatically decrease their growth potential (unpublished data). In spite the absence of colonies formation in semisolid medium, both cell lines further possess unchanged ability of adherent growth on plastic surface under standard cultivation condition and therefore we are convinced, that they fulfill all requirements for being designated as osteosarcoma cell lines.

Migration capacity of osteosarcoma cells was analyzed by a basic wound healing assay and an alternative approach measuring the migration of cells through pores of a cell culture insert, thus minimizing the effect of cell division. In both experiments, migration ability of COS4288 and D-17 cells was superior those of other COS cells. This observation correlates well with the RT-qPCR measured increase in miR-9 and decrease in miR-34a levels in these cells, as overexpression of miR-9 promotes and overexpression of miR-34a inhibits osteosarcoma cell migration [54,55]. We speculate that the slight differences in migration capacities of other COS cells observed between scratch- and cell culture insert-based assay can be attributed to differences in cell plasticity necessary to cross the pores in the insert membrane.

Invasive growth and metastasis formation are the two main indications of cancer progression. Our Matrigel^®^-based cell culture insert invasion assay has shown that all newly isolated cell lines possess the capability to invade through an artificial basement membrane and therefore we assume, that they possess features enabling metastasis formation. As expected, D-17 cells, originating from a lung metastasis revealed the highest invasion index, followed by COS3600B cells [56]. The highly migratory COS4288 cells revealed only moderate invasion index despite the high absolute numbers of invaded cells.

To further characterize isolated cell lines, vimentin, cytokeratin, ALPL, p53, and KPNA2 were analyzed immunohistochemically. As expected, all analyzed cells stained positively for the mesenchymal intermediate filament marker vimentin and negatively for epithelial intermediate marker cytokeratin. These data are congruent with those previously reported regarding D-17 cells and other canine and human osteosarcoma cell lines [13,57,58]. As osteosarcoma are of a mesenchymal origin, the osteosarcoma origin of the isolated COS cells was supported.

Bone is the only connective tissue producing ALPL in dogs and it can therefore be used to differentiate the canine osteosarcoma from other vimentin-positive tumors [59]. All tested cell lines demonstrated positive ALPL staining, which is in accordance with previous observations in osteosarcoma cell lines [12,13,16]. Thus, presence of vimentin combined with ALPL confirmed osteosarcoma origin of the isolated COS cells.

p53 protein, commonly known as the “guardian of the genome” is an extraordinary multifunctional protein involved in cellular DNA integrity protection and regulation in normal and tumor cells [60]. Loss of p53 function or altered functionality through the mutation in the p53 gene is a common feature in cancer cells and has been described in canine osteosarcoma as well [61,62]. Heterogeneity in immunostaining intensity and subcellular localization of p53 in canine osteosarcomas was recently reported by Russel et al. [63]. They found that the majority of tumors showed strong staining intensity, as well as both, cytoplasmatic and nuclear staining pattern. Surprisingly, all our isolated cell lines revealed only nuclear staining pattern with intense immunoreactivity in COS3600B cells, mixed (intense and moderate) immunoreactivity in COS4288 cells and weak immunoreactivity in COS3600, COS4074, and D-17 cells. Differences in observed expression pattern of p53 protein between the current manuscript and data previously published by Russel and coworkers can be attributed to several factors. The choice of antibodies used in these studies might play a role, and without the standardized guidelines accepted between different research teams the uniformity of results cannot be guaranteed. Furthermore, the origin of the analyzed material also plays a substantial role. Whereas in the case of COS cells a cell line (selected cell population derived from an osteosarcoma, cultivated in vitro) was analyzed, original tumor tissue sections were subjected to the analysis by Russel. Considering the short half-life of the wild type (wt) p53 rendering its immunohistochemical detection problematic together with the known wt p53 status in D-17 cells, we can speculate that weak immunoreactivity in COS3600 and COS4074 cells is due to wt p53 status and strong immunoreactivity in COS3600B cells is coupled with its mutated form [64,65,66]. However, detailed DNA sequencing analysis or a p53 functional assay must be performed before indicating the p53 status in these cell lines [67,68].

KPNA2 protein, which is involved in the nucleocytoplasmic transport, is overexpressed in various cancers and associated with poor prognosis [69]. A recent study has shown its applicability for differential diagnosis between osteosarcoma and other malignant bone tumors, with strong expression in various types of osteosarcomas [70]. KPNA2 was detected in all our cells. Strongest immunoreactivity in COS4288 and COS3600B cells, which also showed high migratory and invasive potential, supports the potential of KPNA2 as a marker of poor prognosis.

Due to the successful implementation of immune checkpoint inhibitors into the clinical practice in the recent years, immunotherapy for osteosarcoma has attracted attention as a potential treatment modality for this lethal disease [71,72,73]. Immune checkpoint analysis of CD270 (HVEM), CD274 (PD-L1), and CD276 (B7H3) showed higher expression levels in metastases compared to the parental osteosarcomas, with CD274 as well as CD276 also being associated with poorer patient survival [74,75]. Surprisingly, CD276 expression in D-17 cells originating from an OS metastasis was inferior to those observed in COS cells, which were isolated from parental osteosarcomas. Our analysis only revealed a correlation between CD270 and CD274 expression (high expression in COS3600 and COS4074) but not with CD276, despite the strong correlation between all three markers observed by Cascio [74].

Several studies have confirmed the expression of ERBB2 and MET in human and canine osteosarcoma [76,77,78]. In humans, overexpression of ERBB2 was mostly associated with poor outcome, however some authors dispute this [79,80]. Increased levels of ERBB2 were found in canine osteosarcoma cell lines and tissue samples compared with normal bone [77]. Detected ERBB2 expression in all COS cells together with the expression of immune checkpoint markers make COS cells eligible for immunotherapy-oriented studies. While MET expression did not correlate with overall survival time or disease-free survival time in canine osteosarcoma patients, a significant association with a lymphogenic route of metastasis was observed [81]. In osteosarcoma cell lines, including D-17, siRNA or drug-induced MET inhibition resulted in decreased motility and invasiveness [82]. With the exception of D-17 cells, we found no correlation between MET expression and the migratory or invasive potential of COS cells. However, as D-17 cells harbor mutations in several putative cancer driver genes, including a frameshift mutation in MET, one cannot exclude that the observed overexpression of MET is only one part of a concerted action leading to the high migration and invasion behavior of these cells [66].

Matrix metalloproteinases (MMPs) are proteolytic enzymes with multiple functions in health and diseases [83]. Their role in cancer is closely linked, but not limited, to tumor spread [84,85]. High levels of zymogen precursors of MMP-2 and MMP-9 as well as active MMP-2 were found in most canine tumors, including osteosarcoma [86]. In the current study, MMP-2 mRNA was detected in all cell lines whereas MMP-9 was observed in COS3600B, COS4288, and D-17 cells only. We found no correlation between MMP levels and the invasion capacity of the COS cells, probably due to the missing proteolytic activation of the zymogen form indicated by its mRNA levels [87].

The role of long non-coding RNA MALAT1 in the regulation of multiple signaling pathways in osteosarcoma cells was recently reviewed by Farzaneh [88]. In osteosarcomas, MALAT1 is involved in proliferation and metastasis, vasculogenic mimicry, development and regulation of osteosarcoma stem cells, and drug resistance [17,89,90,91,92,93]. High expression levels observed in COS3600 and COS4074 cells correlated with low proliferation capacity (long doubling time) and negatively correlated with miR-9 expression in these cells. Furthermore, MALAT1 levels correlated with high miR-34a expression in the tested osteosarcoma cells, which was previously also reported for melanoma cells [94]. Despite the variable proliferation capacity of the tested cells, no differences were observed in miR-93 levels as had been expected based on previous data obtained on human osteosarcoma cells [95,96].

The formation of multicellular 3D spheroids represents an important attribute of cancer cell lines, as data obtained from spheroid models are more relevant compared to conventional monolayer (2D) cultures [97,98]. This is particularly important for drug discovery using high throughput screening of new substances, since the data show drug resistance as being significantly different between 2D and 3D cultures [99,100,101] indicting 2D propagation as a significant contributor to its low in vivo effectiveness. Several authors described spheroid formation in canine and human osteosarcoma cells, including D-17 and COS4288 cells, which was confirmed in the present study, and additionally demonstrated for the other COS cells [13,15,26,42,102].

As for the inner spheroid organization, a zonal structure has been proposed based on nutrients, oxygen, and waste transport [103]. For D-17 spheroids, central, intermediate, and outer zones were described, whereas a central and outer zone was described in COS4288 spheroids [26,42]. In the present study, zonal organization could be shown for all analyzed spheroids and confirmed for D-17 and COS4288 cells. The presence of round cells with chondrocyte-like appearance, the high amount of the Alcian-blue-positive extracellular matrix in the central zone, and the absence of calcium deposits in COS4288 spheroids was surprising, as the tumor from which COS4288 cells were isolated was classified as an osteoblastic osteosarcoma. In contrast, although originating from a chondroblastic osteosarcoma, chondroid features were sparse in COS4074 spheroids, but calcium depositions were detectable. However, osteosarcomas feature a high intratumor heterogeneity with many osteosarcomas containing both osteoid and chondroid matrix in various proportions [104]. Most probably, COS4288 cells were isolated from a predominantly chondroblastic area of the tumor, whereas the piece for the histopathological classification originated from an osteoblastic part.

In conclusion, four newly isolated canine osteosarcoma cell lines with different biological characteristics were described. They differ in their growth kinetics, migration/invasion capacities, expression of selected genes and microRNAs related to cell migration, invasion, and cellular checkpoint control, but also possess common attributes of OS cells such as mesenchymal origin, ALPL, p53, and KPNA2 protein expression, and multicellular spheroid formation. We are convinced that these cell lines, reflecting the variation of osteosarcomas, can be a useful model for future in vitro studies to broaden the knowledge of osteosarcoma biology and lead to the development of novel, more effective treatment strategies for osteosarcoma patients.

## Figures and Tables

**Table 1:** STR analysis of the newly isolated canine osteosarcoma cells.

	COS3600	COS3600B	COS4074	COS4288
Amelogenin	Y/X	Y/X	Y/X	X/X
AHT 121	98/100	98/100	96/102	102/106
AHT 137	145/153	153/153	147/147	149/149
AHTH 130	129/129	129/129	119/127	123/129
AHTH 171	219/233	219/233	225/231	223/225
AHTH 260	238/244	238/238	246/246	246/254
AHTK 211	89/91	89/89	89/95	95/95
AHTK 253	288/292	288/292	286/286	284/284
CXX 279	116/124	124/124	126/126	124/124
FH 2054	160/172	160/172	152/156	164/164
FH 2848	232/240	232/232	238/238	238/244
INRA 21	91/101	91/91	95/97	95/95
INU 005	124/124	124/124	124/124	124/124
INU 030	144/150	144/150	144/144	144/144
INU 055	210/210	210/210	210/218	218/218
REN 105 L 03	231/241	241/241	231/233	235/235
REN 162 C 04	200/206	200/206	202/206	200/202
REN 169 D 01	216/218	216/218	216/216	212/216
REN 169 O 18	162/168	168/168	162/172	168/168
REN 247 M 23	272/272	272/272	268/268	268/268
REN 54 P 11	238/238	238/238	232/234	222/226
REN 64 E 19	139/147	139/147	149/149	147/147

## Data Availability

Not applicable.

## Supplementary Materials

The following supporting information can be downloaded at: https://www.mdpi.com/article/10.3390/cells12071026/s1, Table S1: Primers used for the RT-qPCR assay; Table S2: STR analysis of original OS tumors; Table S3: Cell comparison summary at a glance.
