# Supplementary material for: Isolation and Characterization of Novel Canine Osteosarcoma Cell Lines from Chemotherapy-Naïve Patients
Source: Cells. 2023 Mar 27;12(7):1026. doi: 10.3390/cells12071026 (PMC10093184; doi:10.3390/cells12071026)
Supplement: Supplementary file 1 [file cells-12-01026-s001.zip › cells-2263594-supplementary.pdf]

SupplementaryTable S1: Primers used for the RT-qPCR assay

mRNA primers

Accession number*	Gene symbol**	Gene name	Primer sequence (5’ – 3’)	Amplicon size (bp)	*E****	Reference
XM_038664116.1, XM_038664117.1, XM_038664118.1, XM_038664119.1	CD270 (TNFRSF14, HVEM)	TNF receptor superfamily member 14	F: GGGACACGATGTGTGAAGA<br>R: ATGTGCTTCCCGCTGAA	110	0.91	-
XM_038653525.1, XM_038653526.1, XM_038653527.1, XM_038653528.1, XM_038653529.1, XM_038653530.1, XM_038653531.1, XM_038653532.1	CD274 (PD-L1)	CD274 molecule	F: CGCTGAACATCAATGCAACAG<br>R: GTCCTCTCACTTGCTGGAAC	127	1.06	-
XM_038442892.1. XM_038442893.1	CD276 (B7-H3)	CD276 molecule	F: GTGTGCTGGAGGAAGATCAAG<br>R: TGCTTTCAGAGTGTTTCAGAGG	118	0.93	-
XM_038674965.1	ERBB2	erb-b2 receptor tyrosine kinase 2	F: CCCAGAATGGCTCAGTGAC<br>R: CATGAAGGACAGGTCAGGTTT	128	0.93	-
MH050810.1	MALAT1	metastasis associated lung adenocarcinoma transcript 1	F: GCAGTTCGTGGTGAAGATAGGAA<br>R: TCCTAGCTTCACCTCCAAATCG	115	0.97	-
XM_038685904.1, XM_038685905.1, XM_038685906.1, XM_038685907.1, XM_038685908.1	MET	MET proto-oncogene, receptor tyrosine kinase	F: CCTACCATACATGAAACATGGAGA<br>R: ATGCCTTTGGCTACTTGAAGA	108	0.9	-
XM_038659327.1	MMP-2	matrix metalloproteinase 2	F: AGCAAGAACAAGAAGACCTACA<br>R: ATCTGCGATGAGCTTGGG	102	0.97	-
NM_001003219.2	MMP-9	matrix metalloproteinase 9	F: CGCATGACATCTTCCAGTACCA<br>R: CCGAGAATTCACACGCCAGTA	74	0.93	36
NM_001127234.1	OAZ1	ornithine decarboxylase antizyme 1	F: CTGCTGTAGTAACCTGGGTC<br>R: ACATTCAGCCGATTATCAGAGTA	145	0.97	34

XM_853403.4	RPL8	ribosomal protein L8	F: TCTTCCGCCAACAGAGCC<br>R: CTTTGCCTTGTACTTGTGGTAAGC	102	0.94	35
NM_001003102.2	RPL27	ribosomal protein L27	F: ACTACAATCACCTCATGCCC<br>R: CTTGTACCTCTCCTCGAACTTG	143	0.94	34
NM_001252169.1	RPL32	ribosomal protein L32	F: TGGCCATCAGAGTCACCAATC<br>R: GACGCGCACATAAGCTGTTTAT	74	0.94	35

*National Center for Biotechnology Information (NCBI), Entrez Gene [http://www.ncbi.nlm.nih.gov/sites/entrez?db=gene]; **Alternate gene symbols are shown in brackets; ****E*: PCR amplification efficiency; F and R: forward and reverse primer

miRNA and snRNA primers

**miRNA ID***	**Accession ID***	**Forward primer sequence (5' – 3')**	***E*****
cfa-miR-9	MIMAT0006674	GCAGTCTTTGGTTATCTAGCTG	0.98
cfa-miR-34a	MIMAT0006690	GGCAGTGTCTTAGCTGGT	0.98
cfa-miR-93	MIMAT0006696	GCAAAGTGCTGTTCGTG	0.99
cfa-miR-103	MIMAT0006687	GCAGAGCAGCATTGTACAG	1
**Small nuclear RNA*****	**Ensembl ID*****	**Forward primer sequence (5' – 3')**	***E*****
U6 spliceosomal RNA	ENSCAFT00000033247.1	GCTTCGGCAGCACATAT	0.97

*miRBase version 22.1 [https://mirbase.org/]; ***E*: PCR amplification efficiency; ***Ensembl genome browser release 104 [https://www.ensembl.org/]

Supplementary Table S2. STR analysis of original OS tumors

	**COS3600**	**COS4074**	**COS4288**
Amelogenin	Y/X	Y/X	X/X
AHT 121	98/100	96/102	102/106
AHT 137	-/-	147/149	149/149
AHTH 130	129/129	119/127	123/129
AHTH 171	219/233	225/231	223/223
AHTH 260	-/-	-/-	-/-
AHTK 211	91/91	89/95	87/95
AHTK 253	288/292	-/-	284/284
CXX 279	116/124	126/126	124/124
FH 2054	160/172	152/156	164/172
FH 2848	232/240	238/238	238/244
INRA 21	91/101	95/97	-/-
INU 005	124/124	124/124	124/124
INU 030	144/150	144/144	144/150
INU 055	210/210	210/218	218/218
REN 105 L 03	231/241	231/231	235/235
REN 162 C 04	200/206	202/206	200/202
REN 169 D 01	216/218	216/216	212/216
REN 169 O 18	164/168	162/172	168/168
REN 247 M 23	272/272	268/268	268/268
REN 54 P 11	238/238	232/234	222/226
REN 64 E 19	139/147	149/149	147/153

Supplementary Table S3. Cell comparison summary at a glance

cells	**COS3600**	**COS3600B**	**COS4074**	**COS4288**	**D-17**
Cell morphology	spindle-shaped/ seltoid-shaped	spindle-shaped/ cobblestone-like	deltoid-shaped/ polygonal	deltoid-shaped	spindle-shaped
Population doubling time (h)	139	22	426	32	19
Anchorage-independent growth	no	yes	no	yes	yes
invasion index (%)	71	77	46	61	87
ALPL expression	high/low level	high level	high/low level	high level	high/low level
p53 expression	low level	high level	low level	high/moderate level	low level
KPNA2 expression	low level	moderate level	high level	high level	high level
Spheroid formation	yes, two zones	yes, two zones	yes, two zones	yes, two zones	yes, three zones
Von Kossa staining, spheroid	positive	positive	positive	negative	positive
Alcian blue staining, spheroid	negative	weakly positive	weakly positive	positive	negative
Safranin O staining, spheroid	negative	negative	negative	negative	negative

ALPL – alkaline phosphatase, immunodetection
KPNA2 – karyopherin α, immunodetection
